# TRPM7 Ion Channel: Oncogenic Roles and Therapeutic Potential in Breast Cancer

**DOI:** 10.3390/cancers13246322

**Published:** 2021-12-16

**Authors:** Clément Cordier, Natalia Prevarskaya, V’yacheslav Lehen’kyi

**Affiliations:** 1 Laboratory of Cell Physiology, INSERM U1003, Laboratory of Excellence Ion Channels Science and Therapeutics, Department of Biology, Faculty of Science and Technologies, University of Lille, 59650 Villeneuve d’Ascq, France; clement.cordier2.etu@univ-lille.fr (C.C.); natacha.prevarskaya@univ-lille.fr (N.P.); 2 University Lille, Inserm, U1003-PHYCEL-Physiologie Cellulaire, F-59000 Lille, France

**Keywords:** breast cancer, TRPM7, chanzyme, Ca^2+^, therapeutic target

## Abstract

**Simple Summary:**

Breast cancer is the most frequently diagnosed malignant tumor and the second leading cause of cancer death in women worldwide. The risk of developing breast cancer is 12.8%, i.e., 1 in 8 people, and a woman’s risk of dying is approximately 1 in 39. Calcium signals play an important role in various cancers and transport calcium ions may have altered expression in breast cancer, such as the TRPM7 calcium permeant ion channel, where overexpression may be associated with a poor prognosis. This review focuses on the TRPM7 channel, and the oncogenic roles studied so far in breast cancer. The TRPM7 ion channel is suggested as a potential and prospective target in the diagnosis and treatment of breast cancer.

**Abstract:**

The transient receptor potential melastatin-subfamily member 7 (TRPM7) is a divalent cations permeant channel but also has intrinsic serine/threonine kinase activity. It is ubiquitously expressed in normal tissues and studies have indicated that it participates in important physiological and pharmacological processes through its channel-kinase activity, such as calcium/magnesium homeostasis, phosphorylation of proteins involved in embryogenesis or the cellular process. Accumulating evidence has shown that TRPM7 is overexpressed in human pathologies including breast cancer. Breast cancer is the second leading cause of cancer death in women with an incidence rate increase of around 0.5% per year since 2004. The overexpression of TRPM7 may be associated with a poor prognosis in breast cancer patients, so more efforts are needed to research a new therapeutic target. TRPM7 regulates the levels of Ca^2+^, which can alter the signaling pathways involved in survival, cell cycle progression, proliferation, growth, migration, invasion, epithelial-mesenchymal transition and thus determines cell behavior, promoting tumor development. This work provides a complete overview of the TRPM7 ion channel and its main involvements in breast cancer. Special consideration is given to the modulation of the channel as a potential target in breast cancer treatment by inhibition of proliferation, migration and invasion. Taken together, these data suggest the potential exploitation of TRPM7 channel-kinase as a therapeutic target and a diagnostic biomarker.

## 1. Introduction

The mammary glands are glandular organ divided into 15 to 20 lobes containing numerous smaller lobules, which end in dozens of tiny, milk-secreting bulbs. The lobes and lobules are linked together by the milk ducts which collect the milk. Breast cancer can begin in different areas, such as the ducts, the lobules, or the tissue in between, so there are different types of breast cancer, including non-invasive, invasive and metastatic breast cancers [1].

Currently, the risk of developing breast cancer is 12.8%, i.e., one in 8 people [2], and this disease is recognized mainly in people from 30 to 80 years old with a maximum incidence from 50 years old, i.e., postmenopausal women, with 78% of all breast cancers occurring in women of more than 50 years of age and 86% of breast cancer deaths occurring in this age group [3,4]. However, the breast cancer incidence rates have increased slightly since 2004 (by around 0.5% per year), which has been attributed in part to the continued decline in the fertility rate, as well as the increase in obesity. The incidence varies by ethnicity, being highest in industrialized regions, such as Europe and North America (92 per 100,000), and, in contrast, is lower in developing continents such as Africa (27 per 100,000) [5,6].

According to the current prognosis, breast cancer is the most commonly diagnosed malignant tumor and the second leading cause of cancer mortality in women in both industrialized and developing countries [7]. In fact, in the United States, 284,200 new cases are estimated in 2021 and 44,130 deaths, so the risk of a woman dying from breast cancer is about 1 in 39, i.e., 2.6% [4].

Mammography is a technique for detecting breast cancer, which can use density, architectural distortions and the presence of microcalcifications. Moreover, the presence of microcalcifications in a breast tumor has prognostic importance, indeed, many studies highlight links between microcalcifications and high tumor grade [8,9] or significant risk of recurrence [10,11].

Calcium signals play important roles for interacting with the microenvironment and are an essential part of cancer hallmarks. Indeed, breast cancer is intrinsically linked to calcium homeostasis and some of the channels as well as the pumps involved in the transport of calcium ions can have an altered expression in breast cancers, modifying cellular behavior [12]. Certainly, Li et al. demonstrated repeatedly that in patients treated for hypertension or other cardiovascular diseases, with calcium channel blockers (CCB), this would increase the risk, by up to two times, of developing breast cancer but also following a dose long term, which is supported by other studies [13,14,15]. Conversely, more recent studies do not suggest any increased risk of developing breast cancer with long-term or punctual CCB intake, nor is it associated with greater mortality [16,17] and would protect against a risk of recurrence in women aged 20 to 54 [18]. Nevertheless, the use of CCB in breast cancer patients could be associated with the development of lymphedema as well as immunosuppressive effects within the tumor microenvironment [19,20], thus justifying the importance of calcium in breast cancer.

Several studies have investigated TRP channels in human breast cancer, most of which are permeable for Ca^2+^ [21]. In human breast ductal adenocarcinoma tissues (hBDA) and human breast cancer epithelial cells (hBCE), mRNA and protein levels of TRPM7 channel was overexpressed and correlated with high Ki-67 (>10%), a large tumor size (>2 cm) or highly proliferative tumors when compared to the non-tumor tissues [22,23]. Breast cancer patients with an overexpression of TRPM7 have a poor prognosis [24,25,26]. Therefore, a better understanding of expression and oncogenic function of the TRPM7 channel could give rise to a new generation of therapeutic approaches against breast cancer.

In this review, we will present the bulk of current knowledge on the role of the TRPM7 ion channel in the initiation and progression of breast cancer, then as a potential therapeutic target.

## 2. TRPM7 Ion Channel

### 2.1. Structure of Channel

The transient receptor potential (TRP) superfamily of ion channels contains about thirty members in mammals, forming a new family of non-selective cation permeable channels and is organized into seven subgroups according to their sequence homology [27]. The main ones being TRPV for “vanilloid” (TRPV1 to TRPV6), TRPC for “canonical” (TRPC1 to TRPC7) and TRPM for “melastatin” (TRPM1 to TRPM8), most of which are permeable for Ca^2+^, and some also for Mg^2+^ [21,27,28]. These channels, activated in various ways (heat, acidity, stretching, exogenous substances) are mainly in cationic signaling and cause membrane depolarization. Defined as environmental and chemical sensors, they are involved in many physiological functions such as intestinal and renal reabsorption of calcium, mechano-reception, thermoception or even nociception and may participate in tumorigenesis mechanisms [29].

**Figure 1:**
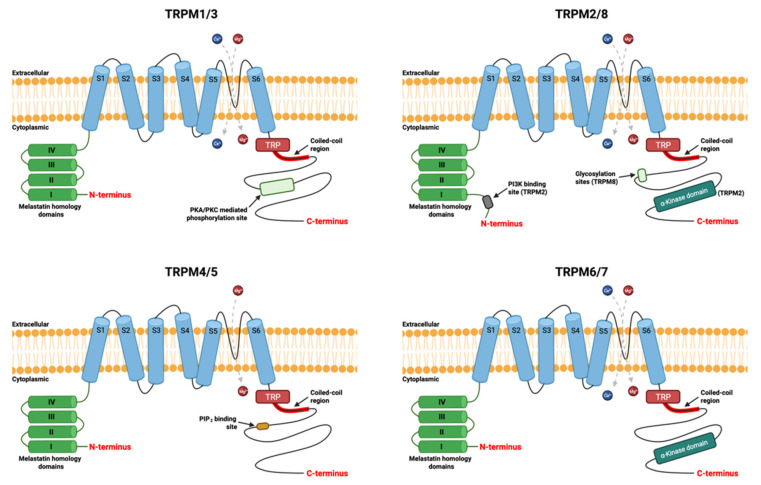
The general structure of subunits of the TRPM ion channel. Each subunits comprises six transmembrane segments (S1 to S6). The channel pore is located between the segments S5 and S6. It is permeable to divalent magnesium (Mg^2+^) and calcium (Ca^2+^) cations, except TRPM4/TRPM5. The amino-terminal (N-terminal) part has homologies’ domains of melastatin members. The carboxy-terminal (C-terminal) is the various part with a coiled-coil region involved in multimerization of the channels. Created using source BioRender.com source, accessed on 9 December 2021.

The transient receptor potential cation channel melastatin-subfamily (TRPM), is a superfamily of non-selective cationic channels, composed of eight members, TRPM1 to TRPM8, involved in various physiological functions ranging from redox detection to insulin secretion, but also in various pathologies such as cancer [30,31,32]. TRPM channels have a large cytosolic domain of between 732 and 1611 amino acids for each subunit and share a TRPM homology domain of about 700 aa in the N-terminal region, a compound transmembrane domain of six transmembrane segments, a quarter pore. However, the C-terminal domain differs among members, varying from 1000 to 2000 aa [33]. This structural variability allows the members of the TRPM subfamily to be divided into four subgroups according to their homologies: TRPM1/TRPM3, TRPM2/TRPM8, TRPM4/TRPM5 and TRPM6/TRPM7 as shown in Figure 1. These channels have different permeabilities to calcium, the TRPM4/5 members being impermeable to calcium and the TRPM6/TRPM7 members being highly permeable to calcium and magnesium [27]. In the next parts, we will focus on TRPM7.

**Figure 2:**
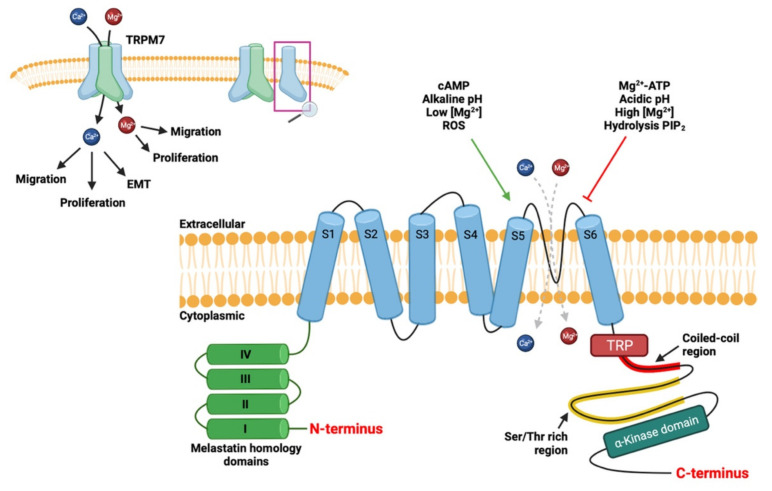
The TRPM7 ion channel is a hetero- or homo-tetramer with each subunit comprising of six transmembrane segments (S1 to S6). The channel pore is located between the segments S5 and S6. It is permeable to divalent magnesium (Mg^2+^) and calcium (Ca^2+^) cations which participate in migration, cell profiling and epithelial-mesenchymal transition (EMT). The amino-terminal (N-terminal) part has four homologies’ domains of melastatin members. The carboxy-terminal (C-terminal) part has a domain common to the channels of the transient receptor potential (TRP), followed by a coiled-coil region involved in multimerization of the channels, then a Serine/Threonine (Ser/Thr) rich region and a terminal COOH α-kinase domain. The kinase domain of TRPM7 has been shown to form a dimer, which can autophosphorylate as well as protein substrates. The channel can be activated by cAMP, alkaline pH, low magnesium concentration, reactive oxygen species (ROS) and inhibited by Mg^2+^-ATP, acidic pH, high magnesium concentration and hydrolysis of PIP_2_. “↑“ means activation and “⊥” means inhibition. Created using source BioRender.com source, accessed on 13 September 2021.

The transient receptor potential cation channel melastatin-subfamily, member 7 (TRPM7, ChaK1, TRP-PLIK, LTRPC7), is ubiquitously expressed in normal tissues [21,34,35,36]. This protein forms a homo- or hetero-tetrameric channel present at the plasma membrane that is highly permeable of the divalent cations like Ca^2+^, Mg^2+^ and Zn^2+^ [27,37], but the selectivity of TRPM7 for Mg^2+^ is higher than for Ca^2+^ [37]. The TRPM7 protein is made up of 6 transmembrane domains, a pore-forming domain between the 5th and 6th transmembrane domains, a coiled-coil domain involved in tetramerization of channels and an COOH-terminal α-kinase domain. It is one of the few to have both ion channel activity and enzymatic activity as shown in Figure 2 [21,38].

TRPM7 is an ion channel and serine/threonine kinase, i.e., a chanzyme with kinase activity regulating the opening probability of the channel [39,40]. In fact, the kinase domain associates directly with phospholipase C (PLC) and more specifically with the C2 domain, allowing PLC to regulate the opening of the TRPM7 channel [39,40,41,42]. Furthermore, activity is also regulated by intracellular levels of Mg^2+^, Mg-ATP and is strongly activated when Mg-ATP levels fall below 1 mM, so TRPM7 is a sensitive channel of the metabolic state of the cell [43,44,45]. Nevertheless, the channel activity can be regulated by other secondary messengers such as PIP_2_, pH, reactive oxygen species and cAMP on Serine residue S1269. [41,46,47,48,49,50].

### 2.2. Physiological Role of TRPM7

The TRPM7 ion channel is involved in various physiological and pharmacological processes both with its channel activity and its kinase activity [47,51]. On the one hand, the activity of the channel plays a central role in calcium homeostasis and in magnesium homeostasis, as shown by the studies of Clark et al as well as Schmitz et al, respectively. In fact, its knockdown effect affects the basal concentration of Ca^2+^ and Calcium “sparks” or “flickers” [52,53,54,55] and pore mutants indicate involvement in cellular homeostasis of Mg^2+^ [40,56]. On the other hand, its kinase domain can phosphorylate several target proteins, involved in embryogenesis [49,51,57,58], cell adhesion and migration [24,52], perception mechanical signals through adhesion sites [59,60,61,62], cell proliferation and viability [43,63], death anoxic cellular [64], neuronal transmission [65] and gastrointestinal stimulation activity [66]. 

Through its varied physiological roles, TRPM7 is of major importance in many human pathologies including various cancers, such as the pancreas, lungs, stomachs, melanoma, prostate and breast. In prostate cancer, the upregulation of TRPM7 expression is involved in increased migration and invasion into cells, indeed as shown in the study by Chen et al, a knockdown of TRPM7 in cells. PCa cells suppress cell migration and invasion, impacting the state of epithelial-mesenchymal transition (EMT), downregulating MMPs, and upregulating E-cadherin [67]. In pancreatic cancer, the same authors demonstrate that a decrease in the expression of this channel via siRNA, leads to an inhibition of cell migration [68]. For gastric cancer, several studies suggest that TRPM7 participates in the survival of human gastric adenocarcinoma cells by acting as detoxifiers [69,70,71]. Each role in different cancers is listed in Table 1.

The TRPM7 ion channel will be involved in several cellular processes in cancer such as viability, cell adhesion or migration and apoptosis. The poor prognosis of breast cancer patients with an overexpression of this channel, raises questions about the role of the channel in the mechanisms of tumor development of breast cancer.

The details of the roles of TRPM7 on breast cell tumorigenesis will be developed in the next sections.

## 3. Role of TRPM7 in Breast Cancer Pathophysiology

Recently, Meng et al, suggest that the expression levels of TRPM7 mRNA are significantly higher in invasive breast ductal carcinoma, invasive lobular mammary carcinoma, invasive ductal and lobular mammary carcinoma, and mixed ductal and lobular mammary carcinoma, compared to tissue control [26], reflecting an involvement of the canal. The roles of TRPM7 involved in breast cancer, are described in the context of the main pathophysiological processes of cancer, such as proliferation, apoptosis, EMT, migration, invasion and microcalcification.

### 3.1. TRPM7 and Proliferation

In studies using human breast carcinoma cell lines, TRPM7 channels play an important role for maintaining proliferation and decreasing cell death [22].

Initially, Guilbert et al, undertook research on the role of TRPM7 in breast cancer and demonstrated that the silencing of TRPM7 with siRNA in breast cancer cells, MCF-7, decreased both the Mg^2+^-inhibited cationic current and the influx of Mn^2+^, resulting in a decrease in the basal concentration of intracellular calcium in the transfected cells. In addition, the reduction of extracellular calcium or the extinction of TRPM7 decreased the growth of MCF-7 cells. This indicates that the TRPM7 calcium channel activity may contribute in the growth of MCF-7 cancer cells via calcium influx [22].

In breast cancer cells MDA-MB-231, AU565 and T47D, a knockout TRPM7, reduced cell growth and the use of 2-aminoethoxydiphenyl borate (2-APB), an inhibitor of TRP channel activity, demonstrates a cell cycle alteration. The percentage of S phase cells increases and there was a corresponding decrease in the G0/G1 phase in all breast cancer cell lines as well as a decrease in G2/M phase in two lasts cell lines. However, a TRPM7 knockout did not alter the cell cycle, testifying a potential compensatory mechanism for the lack of TRPM7 [105]. Moreover, a previous finding indicated that TRPM7 channels mediated the influx of Ca^2+^ and Mg^2+^ and enhanced the activities of S phase cells [106,107].

More recently, in breast cancer cells, MCF-7, Wang et al, demonstrated that TPEN treatment, a zinc ion chelator, inhibited cell growth leading to degradation of MDMX, a p53-associated protein that can inhibit p53 by interaction. However, the ectopic expression of TRPM7 stabilizes MDMX. TRPM7 is therefore involved in Zn^2+^-mediated stabilization of MDMX in cells treated with TPEN [108].

These data demonstrate that TRPM7 is required for breast cancer cell proliferation and tumor growth via modulation of calcium and zinc concentration by channel activity. Nevertheless, the regulatory mechanism of TRPM7 in the cell cycle is still unclear and could involve cell cycle checkpoints.

### 3.2. TRPM7 and Apoptosis

In breast carcinoma, a pro-survival role of TRPM7 channels has been demonstrated [22]. In fact, the TRPM7 channel is permeable to Ca^2+^, which plays an important role in mitochondria-mediated and endoplasmic reticulum-mediated apoptosis.

In breast cancer cells MCF-7, Kim et al, demonstrated the involvement of TRPM7 in apoptosis induced by *Sophorae Radix* and ginsenoside Rd. These herbal drugs, respectively derived from *Sophorae Flavescens Aiton* and Panax ginseng, inhibit the activity of the TRPM7 ion channel, inducing the intrinsic pathway of apoptosis, via depolarization of the mitochondrial membrane and activation of caspase-9 [82,83].

More recently, in other breast cancer cells, MDA-MB-231, inhibition of the channel with NS8593, increases the apoptotic and antiproliferative effects induced by TRAIL, while inhibition of kinase activity with TG100-115 has no effect, suggesting that TRPM7 mediates TRAIL-induced apoptosis via calcium ion fluxes [109]. In addition, downregulation of the activities of the TRPM7 channel could regulate a reduction in the level of c-FLIP_L_ protein promoting the activation of caspase-8, improving apoptosis and decreasing the expression of c-FLIP_S_. This results in TRAIL-induced apoptosis via the formation of death-induced signaling complexes (DISC) [110].

These results highlight the role of TRPM7 in the apoptotic resistance of breast cancer cells and indicate several processes that can induce cell death depending on cell types.

### 3.3. TRPM7 and Epithelial-Mesenchymal Transition

**Figure 3:**
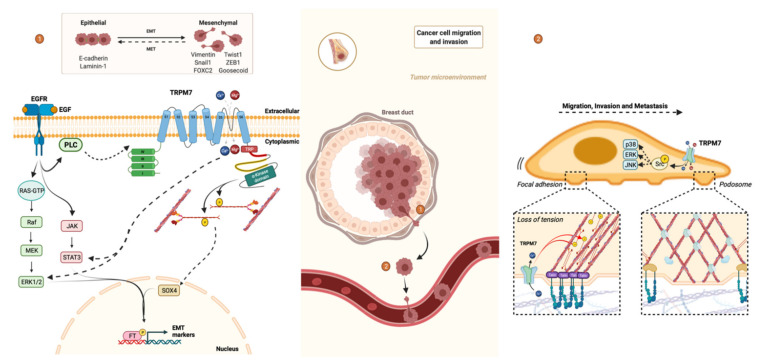
The TRPM7 ion channel is involved in: (1) the epithelial-mesenchymal transition (EMT); and (2) cell migration/invasion in breast carcinoma. Cells must convert their epithelial-like phenotype to mesenchymal-like phenotype in order to acquire a more invasive profile. This involves the loss of cell contacts, modification of the cytoskeleton and increased cell migration and invasion. Breast cancer cells overexpress the TRPM7 channel, a chanzyme. The epidermal growth factor (EGF) allows an increase in the influx of calcium via the EGFR-phospholipase C (PLC) signaling pathway allowing the activation of the channel activity of TRPM7. The elevation of cytosolic calcium induces activation of ERK1/2 and the signal transducer and activator of transcription 3 (STAT3), participating in the expression of vimentin, markers of EMT. The kinase activity of TRPM7 allows phosphorylation (P) of the heavy chain of myosin II (MHC) on the domain responsible for the assembly of myosin filaments, inducing decoupling of the cytoskeleton and cytoskeleton relaxation. Relaxation is associated inversely with regulation by cytoskeletal tension of SRY-Box Transcription Factor 4 (SOX4) expression, participating in the transcription of mesenchymal markers such as Snail1, FOXC2, GSC, SIX1, Twist1, HOXB7 and ZEB1. In addition, MHC phosphorylation by TRPM7 promotes local relaxation of cortical tension and leads to the control of cell-cell adhesion and the transformation of focal adhesions into podosomes, participating in polarized cell movement. The association of TRPM7 with the actomyosin cytoskeleton is Ca^2+^-dependent, which means that the ion influx partly regulates the kinase domain via the interaction with its substrates. TRPM7 is also involved in the phosphorylation of Src and the downstream recruitment of signal molecules such as p38, ERK, JNK, involved in the migration, invasion and metastasis development of breast cancer cells. Created using source BioRender.com source, accessed on 5 October 2021.

Epithelial-mesenchymal transition (EMT) converts epithelial-like phenotype to tumor microenvironment-induced mesenchymal-like phenotype. This results in a more invasive and aggressive phenotype that may participate in resistance to treatment [111]. Concretely, we find the loss of cell contacts, modification of cytoskeletal and increased cell migration and invasion as indicated in Figure 3. This phenotypic switch is important during ontogeny and for pathological processes including the metastasis of solid tumors from the primary site to a secondary organ [112,113].

In human breast cancer cells, MDA-MB-468, epidermal growth factor (EGF)-induced EMT is correlated with a transient elevation of cytosolic Ca^2+^ and accompanied by increased vimentin expression and phosphorylation of signal transducer and activator of transcription 3 (STAT3) markers of EMT. However, silencing of TRPM7 with siRNA or channel inhibitor NS8593, suppresses of the protein level of EGF-induced vimentin and phosphorylation of STAT3 [111].

Nevertheless, TRPM7 does not directly interact with EGF but could play a role downstream to epidermal growth factor receptor (EGFR) in the activation of protein actors such as ERK1/2 and STAT3. EGF could indirectly increase opening probability, i.e., calcium influx through TRPM7 via an EGFR-phospholipase C signaling pathway [111].

In other breast cancer cells, MDA-MB-231, TRPM7 contributes to maintaining a mesenchymal-like phenotype by tensional regulation of the SRY-Box Transcription Factor 4 (SOX4), a recently identified regulator of EMT in breast cancer cells [114]. TRP cation channels, localized within mechano-sensory structures such as cell adhesions, are important transducers of mechanical signals in the tissue homeostasis [115,116]. Therefore, TRPM7 induce cytoskeletal relaxation, through inhibition of myosin II activity by myosin II heavy chain (MHC) phosphorylation via α-kinase domain, is associated inversely with regulation by cytoskeletal tension of SOX4 expression as well as downstream mesenchymal markers such as Snail1, FOXC2, GSC, SIX1, Twist1, HOXB7 and ZEB1. Furthermore, the functional consequences of SOX4 knockdown are similar to TRPM7 knockdown [117].

Thus, the TRPM7 channel is involved in EMT via its participation in the EGF-induced STAT3 phosphorylation and its contribution to the tensional regulation of SOX4.

### 3.4. TRPM7 and Migration/Invasion

Regulation of the cytoskeleton in non-muscle cells play an important role in the regulation of various cellular processes such as cell architecture as seen above, adhesion and migration as indicated in Figure 3 [118,119]. Indeed, myosin II, the main motor protein, interacts with the microfilaments thus forming the actomyosin cytoskeleton responsible for contractility. For this, myosin II assembles in bipolar thick filaments with actin filaments, but the phosphorylation of the MHC tail on threonine residues induces the disassembly of the actomyosin cytoskeleton, leading to a release of cortical tension [120].

In human breast cancer cells, MDA-MB-231, the silencing of TRPM7 with shRNA, alters the cell architecture and decreases the metastatic potential [24]. Indeed, TRPM7 phosphorylates the MHC on the domain responsible for the assembly of myosin filaments [121], thus promoting the local relaxation of cortical tension and leading to the transformation of focal adhesions into podosomes via its kinase domain. However, the phosphorylation of targets by the α-kinase domain is finely regulated by the influx of ions (Ca^2+^, Mg^2+^) through the pore of the TRPM7 channel. The association of TRPM7 with the actomyosin cytoskeleton is Ca^2+^-dependent, meaning that the influx of ions through the pore, partly regulates the kinase domain via the interaction with its substrates [25,52,122].

In other breast cancer cells, MDA-MB-435, a recent study investigated the involvement of Src and mitogen-activated protein kinases (MAPKs) in the migration and invasion of cells. The knockdown of TRPM7 decreases the level of phosphorylation of Src, a regulatory protein that plays a role in cell differentiation, motility and proliferation, as well as the phosphorylation of signal molecules such as p38, ERK and JNK. This is associated with a decrease in the potential for migration and invasion of breast cancer cells [26].

Thus, the TRPM7 channel is involved in the migration and invasion of breast cancer cells via the interaction between its kinase domain and the actomyosin cytoskeleton as well as its contribution to the phosphorylation of Src and MAPKs signaling pathway.

### 3.5. TRPM7 and Metastasis

Metastasis is the leading cause of cancer death and for breast cancer it is correlated with survival and morbidity [123,124]. The formation of metastases is based on several stages including degradation of the extracellular matrix, dissemination of the tumor cell from the primary tumor, intravasation and extravasation through the capillary endothelium, its installation in another tissue site, and finally, the growth of secondary tumors.

Middelbeck et al, provided information on the roles of TRPM7 in tumorigenesis with in vivo studies, using an immunodeficient Rag2^−/−^ IL2rg^−/−^ mouse human breast carcinoma xenograft model. A decrease in the expression of the TRPM7 protein, inducing a decrease in the metastatic potential of human breast cancer cells, indicates a causal effect between the expression levels of TRPM7 and the progression of breast cancer [24]. Moreover, the levels of TRPM7 mRNA are significantly higher in metastatic breast cancers compared to the expression of TRPM7 in primary tumors [125], despite the identical number of copies of the TRPM7 gene [126].

This requires Ca^2+^-dependent remodeling of interactions with the environment such as focal adhesions and adherent junctions resulting from the dynamics of the actomyosin cytoskeleton. Thus, the TRMP7 ion channel mediates focal adhesion number, cell-cell adhesion, and polarized cell movement by regulating MHC phosphorylation [24].

These observations are therefore in agreement with the in vitro studies of the role of TRPM7 in the migration and invasion of cancer cells, as well as the previously announced and confirmed mechanosensory role of TRPM7 in the development of metastases.

### 3.6. TRPM7 and Microcalcification

The presence of microcalcifications in a breast tumor has prognostic importance, between 30% and 50% of non-palpable tumors are found due to the presence of microcalcifications [127]. Many studies highlight links between microcalcifications and malignancy [8,9]. Microcalcifications are composed of hydroxyapatite crystals, or a development of calcium phosphate crystals [128] due to an intracellular accumulation of Ca^2+^ [129]. 

The TRPM7 channel has been shown to be overexpressed in breast carcinoma with associated microcalcifications and it is an important player of Ca^2+^ and Mg^2+^ influx regulation in breast cancer [130].

In human breast cancer cells, MDA-MB-231, the use of 2-APB or silencing of TRPM7 with siRNA significantly decreases intracellular calcium, resulting in total inhibition of mineralization, strongly suggesting a functional role for the channel in the development of hydroxyapatite crystals [131]. However, previous studies have shown TRPM7 to induce a protective effect by promoting an influx of Mg^2+^, an inhibitor of calcium deposition. Similarly to vascular calcification, TRPM7 has an anti-mineralization effect [132,133].

In conclusion, the data demonstrate that TRPM7 via channel activity or kinase activity is a very important player in breast cancer cell aggressivity and ability to develop metastasis.

## 4. TRPM7 as Therapeutic Target in Breast Cancer

As it was shown above, the TRPM7 nonselective cation channel is involved in many oncogenic mechanisms in breast cancer: proliferation, apoptosis, EMT, migration, invasion, metastasis and microcalcifications. Furthermore, for all breast cancer patients in the United States, approximately 15–20% of patients were diagnosed with triple negative breast cancer (TNBC) [134] but, due to the lack of drug targets, such as the estrogen receptor and the progesterone receptor at the cell surface level as in standard treatments, TNBC patients are difficult to cure [135]. This would explain the emergence of novel potent therapies, which target both TRPM7 channel activity and kinase activity, as listed in Table 2.

### 4.1. Impact on Channel Activity

Chemical modulators can inhibit the activity of the TRPM7 channel more or less specifically in breast cancer cells, which can potentially be used in therapeutic applications. They can be used as a local anesthetic in clinics (Lidocaine), natural compounds (Carvacrol, Waixenicin A, Ginsenoside Rd, *Sophorae Radix*) or synthetic compounds (NS8593).

#### 4.1.1. Lidocaine

Lidocaine is the most commonly used local anesthetic and some studies indicate improved clinical outcome of cancer progression after surgery [141]. Indeed, it seems to have direct effects by inhibiting the viability and migration of cancer cells [136]. Additionally, previous studies have suggested that lidocaine blocks TRPM7 channels [142].

In human breast cancer cells, AU565, T47D, MDA-MB-231 and MDA-MB-468, the addition of lidocaine at concentrations ≥0.3 mM, has a suppressive effect on viability as well as migration cellular, involving a decrease in the influx of Mn^2+^, so lidocaine would have a general effect, independent of cell lines, but with a different potency [137].

This confirms the role of TRPM7 as a potential target upstream of lidocaine in the viability and migration of breast cancer cells, with a greater effect in the MDA-MB-231 cell line.

#### 4.1.2. Carvacrol

Carvacrol is a natural bioactive monoterpenoid phenol, which can be used for antifungal, antiviral, anticancer treatment and the regulation of inflammatory activities [143]. In addition, carvacrol is a non-specific inhibitor of the transient potential of TRPM7 channels.

In breast cancer cell lines MDA-MB-231 and MCF-7, the addition of carvacrol inhibits TRPM7 currents, increases cell arrest in G0/G1 phase, and decreases cells in the S and G2/M phase, with different sensitivity. This modulator inhibits cell proliferation by interacting with the cell cycle. However, additional studies remain necessary because the presence of carvacrol modulates the expression of various cyclins and CDK4, while the silencing of TRMP7 does not affect these proteins. Finally, at high concentration, carvacrol induces apoptosis in all breast cancer cell lines with the same potency [138].

Therefore, the TRPM7 pathway could be one of the pharmacological mechanisms of the effect of carvacrol, but the direct or indirect link remains to be determined.

#### 4.1.3. Waixenicin A

Waixenicin A is a xenican diterpenoid isolated from the Hawaiian soft coral *S. edmondsoni* and a potent and relatively specific inhibitor of TRPM7 ion channels and more specifically of the Mg^2+^ current [93].

In breast cancer cell line MCF-7, the presence of 50 μM of waixenicin A in the cell medium halves the number of surviving cells, depending on the intracellular concentration of Mg^2+^. In addition, the presence of this chemical modulator causes cells to stop in the G0/G1 phase [139].

This suggests that waixenicin A could be considered as a potential drug for the pharmacological treatment of breast cancer, by modulating survival and cell cycle progression.

#### 4.1.4. Ginsenoside Rd

Ginsenoside Rd (G-Rd) is known to be one of the most active ginsenosides. The molecular components responsible for the action, ginseng has anti-inflammatory, vasorelaxants, antioxidant and anticancer properties [144].

In breast cancer cell line MCF-7, G-Rd inhibits the activity of the TRPM7 channel, inducing a decrease in cell proliferation and survival as well as activation of apoptosis by apoptotic mechanisms of the intrinsic pathway [82].

#### 4.1.5. Sophorae Radix

Investigated by Kim et al, *Sophorae Radix* (SR) is the dried root of *Sophorae Flavescens Aiton* and has several activities such as antioxidant, antibacterial, anti-inflammatory, antipyretic. [57,145].

In the breast cancer cell line MCF-7, like G-Rd, SR blocks the activity of the TRPM7 channel and activates apoptosis by the apoptotic mechanisms of the intrinsic pathway, inhibiting cell growth and cell survival [83].

There is a need to deepen the mechanism of action of these two previous modulators by increasing the diversity of breast cancer cell lines.

#### 4.1.6. NS8593

The development of a small synthetic compound such as N-[(1R)-1,2,3,4-tetrahydronaphthalene-1-yl]-1H-benzimidazol-2-amine or NS8593 makes it possible to specifically modulate the channels activity of the TRPM7.

Indeed, in breast cancer cell lines MDA-MD-231 and MDA-MD-468 (TNBC cells), NS8593 suppresses TRPM7 Mg^2+^-dependent currents [140], increasing the apoptotic and antiproliferative effects induced by TRAIL via a decrease in c-FLIP as well as suppressing the phosphorylation of STAT3, respectively [109,111].

### 4.2. Impact on Kinase Activity

The TRPM7 ion channel is a chanzyme and its kinase activity is involved in processes of tumorogenesis in breast cancer. Therefore, the development of a molecule inhibiting its α-kinase domain could be a therapeutic hope.

TG100-115, a potent but non-specific inhibitor of the α-kinase domain of TRPM7, is a peptide of CREB. It acts competitively with ATP.

As previously established, the kinase activity of TRPM7 plays a role in the opening of the channel but is also involved in the phenomenon of migration and metastatic development. In breast cancer cell MDA-MB-231, the addition of TG100-115 inhibits the channel activity of TRPM7 and strongly decreases cell migration and invasion, resulting from an inhibition of phosphorylation of the heavy chain of myosin IIA, in this last case.

Therefore, TG100-115 could be used as an inhibitor of the kinase activity of TRPM7 and an inhibitor of the migration of breast cancer cells [122].

This suggests the potential of developing modulators of TRPM7 into anti-breast cancer therapeutics. Nevertheless, TRPM7 is a highly selective channel for calcium and magnesium and is expressed ubiquitously, it is therefore necessary to develop chemical modulators targeting TRPM7 locally, thus, avoiding as much as possible, the side effects that could be harmful for the patient.

## 5. General Conclusions and Perspectives

Throughout this review, the importance of TRPM7 ion channels in the oncogenic phenomenon in breast cancer cells has been investigated. Initially, TRPM7 is a ubiquitously expressed divalent cation channel with intrinsic serine/threonine kinase activity that plays a role in a variety of cellular processes such as calcium/magnesium homeostasis, embryogenesis, neuronal transmission and human pathologies, including various cancers. Several studies report overexpression of channels in breast cancer cells, and experimental evidence implicates important roles of the TRPM7 chanzyme as an important player in the aggressiveness of breast cancer cells. Its channel activity is involved in cell proliferation and tumor growth via modulation of calcium concentration, in apoptotic resistance via calcium flux, in epithelial-mesenchymal transition and in the development of microcalcification. Its serine/threonine kinase activity is also involved in EMT, migration and metastatic development. Future studies are indicated to improve the understanding of the role of the TRPM7 channel kinase in the characteristics of cancer and more specifically the targets of its kinase activity as well as its direct implications in the development of breast cancer.

Breast cancer is still considered the second leading cause of cancer death in women, and despite the poor prognosis associated with overexpression of TRPM7, no effective treatment exists to date. Standard care treatments only affect the general tumor, and diagnostic markers still lack sensitivity. Therefore, the expression of TRPM7 may constitute a clinical biomarker for the prevention and early detection of breast cancer, as well as a therapeutic target for new treatments but animal models are needed. Targeting the TRPM7 channel may therefore represent an important prospect for the foreseeable future.

## Figures and Tables

**Table 1:** Expression and oncogenic roles of TRPM7 in cancers.

Involved Cancer	Expression	Oncogenic Roles	Ref.
Bladder	○ Over-expressed in MBT-2 cells	○ Knockdown of TRPM7 prevents tumor growth, proliferation, migration and invasion via the Src, Akt and JNK pathway ○ Decreased TRPM7 induces apoptosis via ERK1/2 pathway	[72,73,74]
Breast	○ Over-expressed in human breast carcinoma tissues and cell lines	○ Detailed in this review	Not reported
Cervical	○ Over-expressed in cell lines	○ miR-543 inhibits tumor growth and metastasis by targeting TRPM7 ○ miR-192-5p inhibits proliferation and invasion by targeting TRPM7 ○ Involved in acidotoxic necrotic cell death	[75,76,77]
Colorectal	○ Over-expressed in human IBD-related and sporadic colorectal cancer ○ Over-expressed in TRPM7 expression in CRC tissues	○ Decreased TRPM7 in vitro inhibits cell proliferation, migration and invasion by reversing EMT status ○ Decreased TRPM7 in vitro induces apoptosis	[78,79,80]
Erythroleukemia	○ TRPM7-like currents in cell lines	○ Not reported	[81]
Gastric	○ Over-expressed in human gastric adenocarcinoma cell lines ○ Somatic mutation M830V in gastric adenocarcinoma	○ Involved in cell survival via Mg^2+^ ○ Waixenicin A inhibits growth and survival of cancer cells	[69,82,83,84]
Glioblastoma	○ Over-expressed in human glioblastoma patients	○ Involved in proliferation, migration and invasion of glioma cells via JAK2/STAT3 and/or Notch signaling pathways ○ Waixenicin A inhibits proliferation, migration, invasion and survival of cancer cells ○ Involved in vesicular transfer of CLIC1 from glioblastoma to microvascular endothelial cells	[85,86,87,88,89]
Head and Neck carcinoma	○ Expressed in FaDu cells and SCC-25 Cells ○ Over-expressed in 5-8F cells and low expression in 6-10B cells	○ Involved in growth and proliferation of cancer cell ○ Involved in migration of nasopharyngal carcinoma cells ○ Involved in activation of AKT/mTOR signaling pathways, which could act as a risk factor for the progression of HNC	[90,91]
Leukemia	○ Expressed in Leukemia K562 cells	○ Waixenicin A inhibits proliferation of cancer cells ○ Involved in hemin-induced erythroid differentiation of a human erythromyeloid leukemia cell line K562	[92,93]
Lung	○ Expressed in A549 cells ○ Expressed in lung adenocarcinoma and squamous cell lung carcinoma	○ Involved in tumor cell motility and metastasis via c-Myc in lung carcinoma ○ Involved in migration of A549 cells via EGF	[94,95]
Melanoma	○ Expressed in cell lines	○ Not reported	[96]
Neuroblastoma	○ Not reported	○ Involved in formation of Ca^2+^ sparking and invadosome by affecting actomyosin contractility in N1E-115 cells	[55]
Pancreatic Adenocarcinoma	○ Over-expressed in human tissues cell lines	○ Involved in proliferation, cell cycle progression, tumor growth, invasion and metastasis formation of cancer cells via Mg^2+^ ○ Involved in migration in PDAC via TRPM7/RPSA complex ○ Prevents replicative senescence of cancer cells	[97,98,99]
Prostate	○ Over-expressed in human prostate cancer cell lines	○ Involved in apoptosis of PC-3 cells induced by TRAIL ○ Induced in proliferation, migration and viability of prostate cancer cells ○ Knockdown of TRPM7 induces a reverse the epithelial-mesenchymal transition (EMT) status	[67,100,101,102]
Ovarian	○ Over-expressed in ovarian carcinoma ○ Somatic mutation S406C in ovarian serous carcinoma	○ Involved in proliferation, migration, invasion and cancer metastasis ○ Knockdown of TRPM7 decreases phosphorylation levels of Akt, Src and p38 and increases focal adhesion number of cancer cells	[77,103,104]
Retinoblastoma	○ Expressed in 5–8F cells	○ Involved in proliferation and migration of cancer cell	[63]

**Table 2:** Chemical modulators of TRPM7 channel activities as potential anticancer therapies.

Impacted Activity	Chemical Modulator	Mechanism of Action	Ref.
Channel Activity	Lidocaine	○ Inhibits TRPM7 currents ○ Inhibits the viability and migration of cancer cells ○ Decrease in the influx of Mn^2+^	[136,137]
Channel Activity	Carvacrol	○ Inhibits TRPM7 currents ○ Increases cell arrest in G0/G1 phase and decreases cells in S and G2/M phase ○ Inhibits cell proliferation by interacting with the cell cycle ○ High concentration induces apoptosis	[138]
Channel Activity	Waixenicin A	○ Inhibits Mg^2+^ current ○ Decreases the number of surviving cells ○ Increases cell arrest in the G0/G1 phase	[93,139]
Channel Activity	Ginsenoside Rd	○ Decreases cell proliferation and survival ○ Activates apoptosis by apoptotic mechanisms of the intrinsic pathway	[82]
Channel Activity	Sophorae Radix	○ Inhibits cell growth and cell survival ○ Activates apoptosis by apoptotic mechanisms of the intrinsic pathway	[83]
Channel Activity	NS8593	○ Increases the apoptotic and antiproliferative effects induced by TRAIL	[109,111,140]
Kinase Activity	TG100-115	○ Inhibits the channel activity ○ Decreases cell migration and invasion: inhibition of phosphorylation of the heavy chain of myosin IIA	[122]
